# Case report: Germline *CHEK2* mutation is associated with a giant cell glioblastoma

**DOI:** 10.3389/fonc.2024.1361928

**Published:** 2024-10-01

**Authors:** Yongfeng Bi, Dong Wan, Si Chen, Huafei Chen, Lingchuan Guo, Xiaoshun He, Rong Rong, Jinyuan Xiao, Wei Gao, Sheng Xiao

**Affiliations:** ^1^ Department of Neurosurgery, The First Affiliated Hospital of Soochow University, Suzhou, China; ^2^ Advanced Molecular Pathology Institute of Soochow University and SANO, Suzhou, China; ^3^ Sano Precision Medicine Ltd., Suzhou, China; ^4^ Department of Pathology, The First Affiliated Hospital of Soochow University, Suzhou, China; ^5^ Department of Biological Sciences, Xi An Jiaotong-Liverpool University, Suzhou, China; ^6^ The College of Informatics, Huazhong Agricultural University, Wuhan, China; ^7^ Department of Pathology, Brigham and Women's Hospital, Harvard Medical School, Boston, MA, United States

**Keywords:** *CHEK2*, glioblastoma, haploidy, MET, germline

## Abstract

Giant cell glioblastoma often exhibits genome instability and is frequently associated with mutations in genes involved in DNA repair pathways including *TP53* and DNA mismatch repair genes. Several germline mutations have been identified in giant cell glioblastoma, including mutations of *MSH1* and *MSH2*, *TP53*, and *POLE*. We have documented a case of a germline mutation in *CHEK2*, another gene crucial to DNA repair, in a patient with giant cell glioblastoma. The *CHEK2* mutation was inherited from the patient’s father, who had a history of gastric cancer and renal cell carcinoma. In addition to the germline *CHEK2* mutation, the giant cell glioblastoma exhibited a genome-wide loss of heterozygosity, a characteristic observed in a subset of giant cell glioblastomas. Additional mutations detected in the tumor included *TP53, PTEN*, and a *PTPRZ1-MET* fusion. This represents the first reported case of a *CHEK2* germline mutation in giant cell glioblastoma, further supporting the significance of impaired DNA repair mechanisms in the development of this disease.

## Introduction

Giant cell glioblastoma is a rare subtype of glioblastoma characterized by the presence of numerous large and morphologically bizarre tumor cells. Clinically, giant cell glioblastoma tends to have a better prognosis compared to classic glioblastoma. The genomic profile of giant cell glioblastoma differs from that of classic glioblastoma, which likely contributes to its distinct clinical behavior. While classic glioblastoma commonly exhibits a combination of *TERT* mutation, *EGFR* amplification, *CDKN2A/B* loss, and +7/−10 chromosome copy-number alterations, giant cell glioblastoma often shows *TP53* and *PTEN* mutations, with *TP53* mutation being the most frequently observed alteration, present in 90% of cases. Recently, a subgroup of giant cell glioblastoma with a near-haploid genome has been identified (4).

Germline mutations in several DNA repair-related genes, including *TP53, POLE*, and DNA mismatch repair genes *MSH1* and *MLH2* (1–3), have been associated with giant cell glioblastoma, indicating the significance of DNA repair deficiency in the development of this tumor subtype. Here, we present a case involving a germline mutation in the *CHEK2* gene, which is also involved in the DNA damage response pathway. CHEK2 is part of the ATM-CHEK2-p53 axis, which plays a crucial role in the cellular response to DNA damage. Upon detection of DNA damage by ATM, CHEK2 is activated and phosphorylates p53, leading to cell cycle arrest for DNA repair or apoptosis to prevent malignant transformation.

Germline mutations in *TP53* are associated with Li-Fraumeni syndrome, characterized by an increased risk of various cancers, including brain tumors, breast cancer, leukemia, sarcoma, adrenocortical carcinoma, and colon cancer (5). Some individuals with Li-Fraumeni syndrome do not have *TP53* mutations but harbor other mutations, including *CHEK2* mutations. In our patient, who has giant cell glioblastoma, the *CHEK2* mutation was inherited from their father, who had gastric cancer and renal cell carcinoma. The *CHEK2* p.H371Y mutation, located in the kinase domain, results in reduced kinase activity in functional assays [PMID: 21618645] and is associated with breast cancer [PMID: 24390236]. Additionally, breast cancer patients carrying the *CHEK2* p.H371Y mutation are more likely to respond to neoadjuvant chemotherapy compared to non-carriers [PMID: 25884806]. To the best of our knowledge, this is the first documented case of a germline *CHEK2* mutation in giant cell glioblastoma, thereby providing additional evidence for the importance of compromised DNA repair mechanisms in the development of this disease.

## Materials and methods

### Immunohistochemical studies

Immunohistochemistry (IHC) was performed on 5-μm tissue sections using the following protocol: (1) the slides were baked at 60 °C for 1 hour (2), deparaffinized and rehydrated with 100% xylene, 100% ethanol, 70% ethanol, and running water (3), blocked in a solution of 10% normal serum and 1% bovine serum albumin (BSA) in Tris-buffered saline (4), incubated with primary antibodies for 2 hours (5), endogenous peroxidase was blocked with 0.3% hydrogen peroxide, and (6) slides were incubated with horseradish peroxidase-labeled polymer (DAKO) according to the manufacturer’s instructions. The tissue sections were developed using 3,3’-diaminobenzidine (DAKO) as the chromogen and counterstained with Mayer’s hematoxylin.

Antibodies and their working concentrations are listed in **Supplementary Table 1**.

### Targeted DNA next-generation sequencing (NGS)

DNA was extracted from Formalin-Fixed Paraffin-Embedded (FFPE) tumor tissue and peripheral blood, fragmented with a Bioruptor Pico (Diagenode, Denville, NJ) to 200-300 bp, subjected to end-polishing, phosphorylation, and DNA extension by incubating with the end-repair mix, Klenow exo- and Taq polymerase (Enzymatics, Beverly, MA) for 15 min at 12°C, 15 min at 37°C, and 15 min at 72°C, and ligated to a UMl-containing adaptor. Four cycles of polymerase chain reaction (PCR) were performed with adaptor-specific primers and the PCR products were incubated with a pool of biotin-labeled bait oligos targeting 638 genes commonly involved in tumors for 16 h. Targeted regions were enriched by pull-down with streptavidin beads, amplified by PCR and sequenced in an illumina NovaSeq sequencer (San Diego, CA, USA). Sequencing results were analyzed with SeqNext software (JSI, Ettenheim, Germany).

### Targeted RNA sequencing and nested RT-PCR

Total RNA was extracted from fresh tumor sample using the TRIZOL reagent (ThermoFisher, Waltham, MA) according to the manufacturer’s instructions.

The reverse transcription, end repairing, dA-tailing, and adaptor ligation were performed following standard NGS protocols (NEB, Cat E7771 and E6111, Ipswich, MA, USA). A group of 60 genes commonly involved in solid tumors was targeted using PCR enrichment with primers specific to these genes. The PCR products were sequenced using an Illumina NovaSeq sequencer. The sequencing results were analyzed using SeqNext software and laboratory-developed pipelines (Sano Medical Laboratories, China).

For nested RT-PCR, cDNA synthesis from total RNA was performed using random priming and the SuperScript™ IV reverse transcriptase (ThermoFisher). PCR amplification was then carried out using specific primers for PTPRZ1::MET (forward primer PTPRZ1: 5’-CACTCTGAGAAGCAGAGGAGCC and reverse primer MET: 5’-GGTGTTTCCGCGGTGAAGTT; nested PCR forward primer PTPRZ1: 5’-CCGTCTGGAAATGCGAATCC and reverse primer MET: 5’-ATTGCTCCTCTGCACCAAGG). The PCR was performed with one cycle at 95°C for 3 minutes followed by 30 cycles at 95°C for 30 seconds, 60°C for 1 minute and 72°C for 1 minute. The initial PCR product (0.1 µl) served as the template DNA for the subsequent nested PCR. This nested PCR comprised an initial cycle at 95°C for 3 minutes followed by 30 cycles at 95°C for 30 seconds, 58°C for 1 minute, and 72°C for 1 minute. The PCR products were then subjected to Sanger sequencing.

### *MGMT* methylation analysis

The CpG islands of the *MGMT* promoter region from tumor DNA was amplified by using FAM fluorescently labeled primers (forward primer 5’- TTTGTGTTTTGATGTTTGTAGGTTTTTGT, reverse primer 5’- TTTCGACGTTCGTAGGTTTTCGC). The PCR protocol consisted of one cycle at 95°C for 10 minutes followed by 40 cycles at 95°C for 45 seconds, 60°C for 45 seconds and 72°C for 1 minute. The FAM-labeled PCR products were analyzed for fragments in a capillary electrophoresis instrument. Control groups included gDNA specimens known to be methylated and unmethylated at the MGMT promoter.

This study was approved by the institutional review board at respective institutions.

## Results

**Figure 1:**
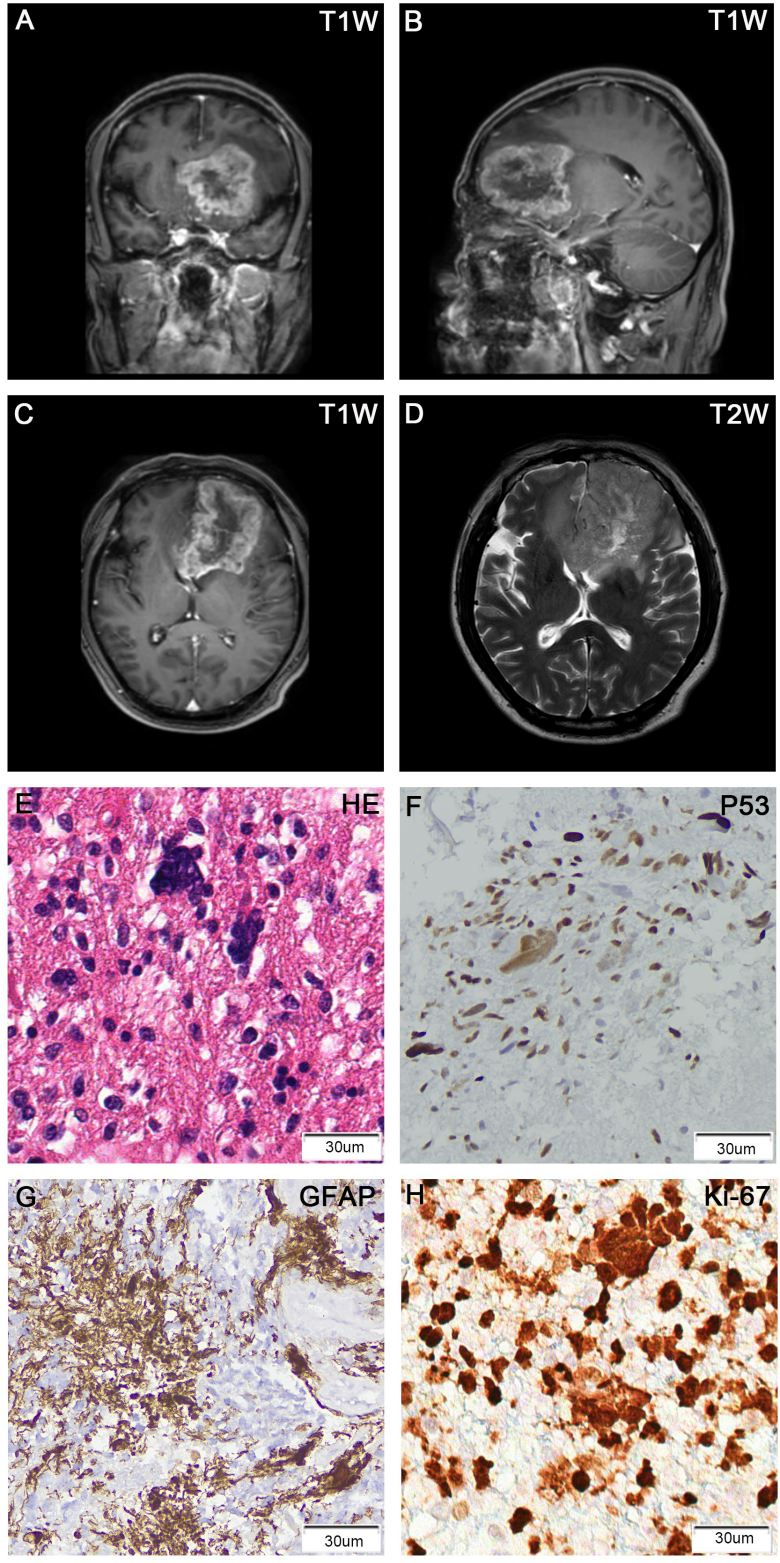
Brain MRI showed a ring-enhancing tumor in the left frontal lobe, with the largest cross-section of 75 x 50 mm. **(A)** T1-weighted contrast-enhanced (coronal view); **(B)** T1-weighted contrast-enhanced (sagittal view); **(C)** T1-weighted contrast-enhanced (axial view); **(D)** T2-weighted (axial view). H&E stain of FFPE tumor sections showed numerous large and multinucleated tumor cells **(E)**. IHC of tumors cells was positive for p53 **(F)**, GFAP **(G)**, and Ki-67 **(H)**.

A 53-year-old male presented with symptoms of delirium, abnormal behavior, and unresponsiveness. Magnetic Resonance Imaging (MRI) revealed a 75 x 50 mm irregular abnormal signal in the left temporal lobe. T1-weighted images showed hypo-to-isointense signals, while T2-weighted images displayed heterogeneous hyperintense signals. Diffusion-weighted imaging (DWI) indicated diffuse restriction, with surrounding edema and ring enhancement (**Figures 1A–D**). These radiological findings were suggestive of glioblastoma. A surgical procedure successfully removed a tumor measuring 50 x 60 x 70 mm. Histopathological analysis of the FFPE tumor tissue section revealed the presence of pleomorphic cells with numerous bizarre multinucleated giant cells and frequent mitotic figures. Immunohistochemical staining showed positive results for GFAP, Olig-2, MGMT, MAP2, ATRX, P53, EGFR, S100, focal vimentin and negative for IDH-1, NeuN, H3K27M, Syn, and CD34. Ki-67 staining demonstrated a high proliferation index of 60% (**Figures 1E–H**; **Supplementary Figure 1**). Based on these findings, a diagnosis of giant cell glioblastoma was made.

**Figure 2:**
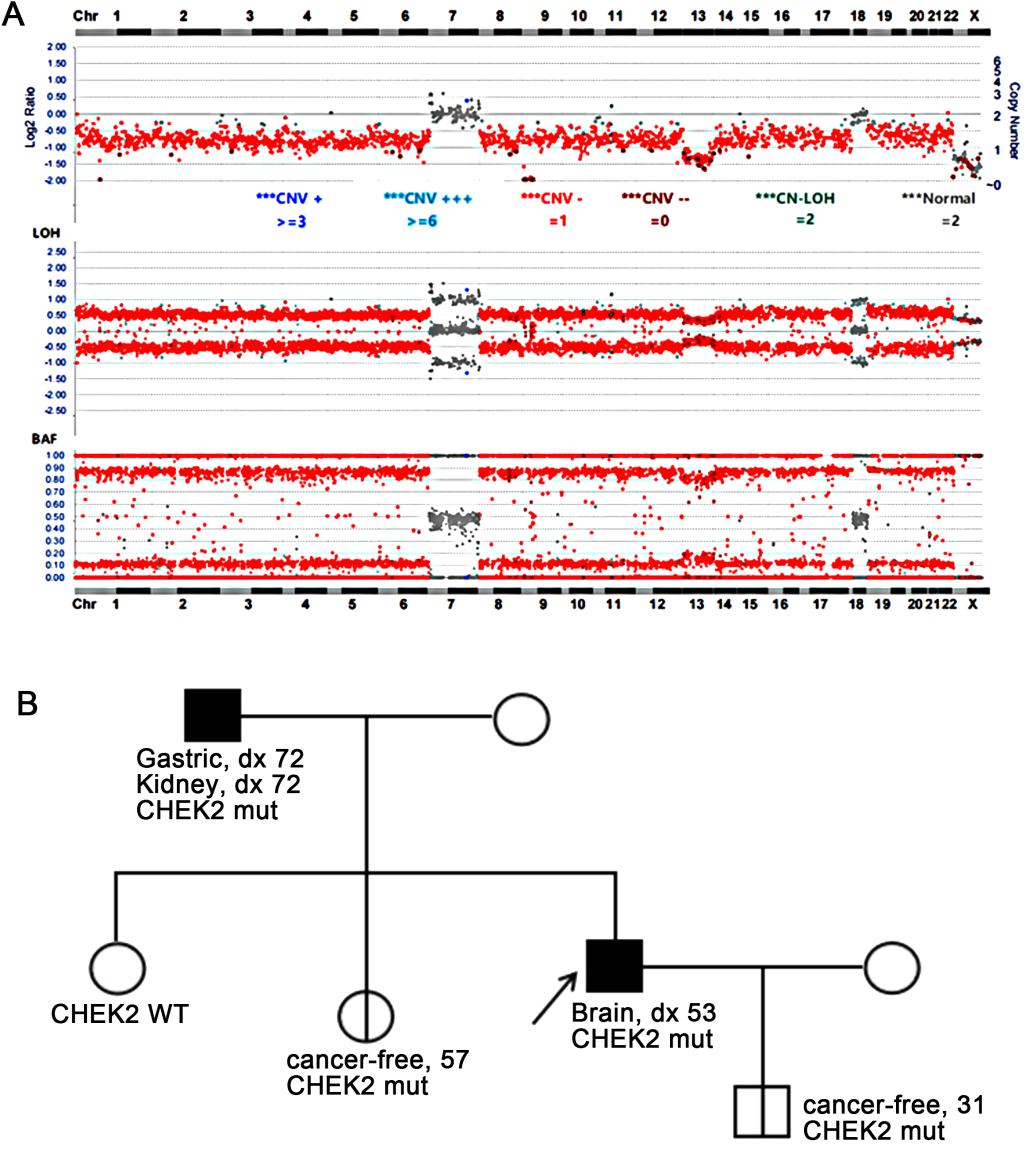
**(A)** Targeted DNA NGS revealed a near-haploid genome with genome-wide loss of heterozygosity, except for chromosomes 7 and 18. Additionally, a homozygous loss of chromosome 13 was observed. **(B)** The family pedigree showed the presence of the germline *CHEK2* p.H371Y mutation in the patient (proband) as well as in his father, one of his sisters, and his son. The father was diagnosed with gastric cancer and renal cell carcinoma at the age of 72, the proband with giant cell glioblastoma at the age of 53, while no tumors were reported in the son at age 31 and the sister at age 57. WT, wild-type; mut, mutation.

Targeted DNA NGS of 638 cancer genes coupled with whole-genome single-nucleotide polymorphism (SNP) analysis was performed on tumor DNA. The results revealed a near-haploid genome with widespread loss of heterozygosity throughout the genome, except for chromosomes 7 and 18, which maintained heterozygosity. Notably, both copies of chromosome 13 were lost (**Figure 2A**). Additionally, three mutations were detected: *TP53* p.T125K, *PTEN* p.D52M fs*2, and *CHEK2* p.H371Y. While the *TP53* and *PTEN* mutations were identified as somatic mutations, the *CHEK2* mutation was also observed in the patient’s peripheral blood specimen, confirming a germline *CHEK2* mutation.

The patient’s family history revealed that the patient’s father had a history of gastric cancer and renal cell carcinoma, both diagnosed at the age of 72. Subsequent *CHEK2* mutation screening in the family confirmed that the patient’s father carried the same *CHEK2* mutation. Additionally, the patient’s sister and son were found to carry the same *CHEK2* mutation, although they have not reported any tumors at the ages of 57 and 31, respectively (**Figure 2B**).

**Figure 3:**
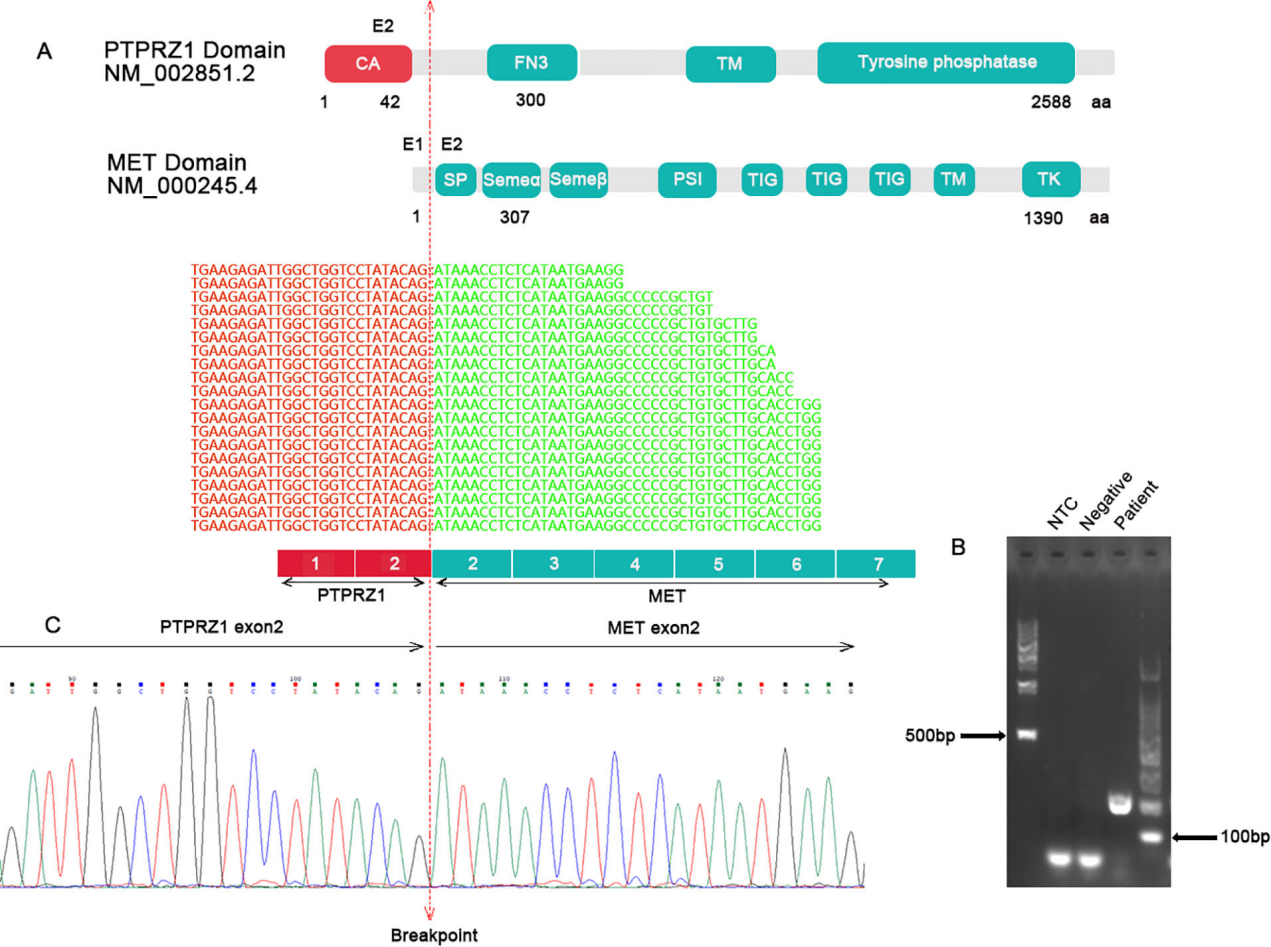
**(A)** Targeted RNA NGS showed an in-frame fusion between *PTPRZ1* exon 2 and *MET* exon 2. The schematic of functional domains of PTPRZ1 and MET and their exon location were shown. The breakpoint was marked by a vertical dotted line. **(B)** RT-PCR with specific primers for *PTPRZ1* and *MET* produced a band at expected size for the *PTPRZ1::MET* rearrangement. **(C)** Sanger sequencing of the PCR product confirmed the *PTPRZ1::MET* fusion. CA, alpha-carbonic anhydrase; FN3, fibronectin-like domain; TM, transmembrane domain; SP, signal peptide; SEMA, Semaphorin domain; PSI, Plexin semaphorin domain; TIG like plexins transcription factor; TM, transmembrane region; TK, tyrosine kinase.

Further analysis was conducted using targeted RNA NGS on a fresh tumor sample, which identified a *PTPRZ1-MET* rearrangement. Specifically, exon 2 of *PTPRZ1* was fused with exon 2 of *MET*, resulting in a chimeric protein with an intact reading frame (**Figure 3A**). The chimeric protein retained a partial alpha-carbonic anhydrase domain from *PTPRZ1* and all functional domains from *MET*, similar to previously described rearrangements (6). The *PTPRZ1-MET* fusion was confirmed an RT-PCR assay using primers specific to *PTPRZ1* and *MET* followed by Sanger sequencing (**Figures 3B, C**). Methylation-specific PCR (MSP) and capillary electrophoresis were performed by evaluating *MGMT* promoter CpG methylation. The results of this analysis demonstrated methylated *MGMT* status (**Supplementary Figure 2**).

Two cycles of temozolomide (TMZ) therapy and one cycle of external beam radiation therapy (EBRT) were administered. Unfortunately, the patient did not respond to these treatments and succumbed to the disease 10 months after the initial diagnosis.

## Discussion

The presence of numerous giant cells is considered a classification-defining feature for giant cell glioblastoma. Giant cells can be seen in various physiological and pathological conditions. The multinucleated osteoblasts, macrophages, and muscle cells are formed by cell fusion. The hepatocytes and trophoblasts during placental development are cells of endoduplication, i.e., cells with repeated rounds of DNA replication without subsequent cell division. Giant cells can be observed in various types of tumors. Alongside giant cell glioblastoma, they are also commonly found in other tumors including bone tumors such as giant cell tumor of bone, chondroblastoma, and aneurysmal bone cyst (7–9). Additionally, giant cells can be present in soft tissue tumors such as dedifferentiated liposarcoma and undifferentiated pleomorphic sarcoma (10, 11). Furthermore, the Hodgkin and Reed-Sternberg cells are a characteristic feature of Hodgkin’s lymphoma (12). The mechanisms underlying giant cell formation in malignancies are likely disease-specific. In the case of giant cell glioblastoma, DNA repair deficiency and genome instability may play a role. DNA repair mechanisms are tightly linked to cell cycle checkpoints, which ensure proper DNA replication and repair prior to cell division. If these checkpoints fail due to DNA repair deficiencies, cells with damaged DNA can proceed through the cell cycle and divide, resulting in abnormal chromosome numbers, including polyploidy. Additionally, DNA repair defects can disrupt the normal process of cytokinesis, the division of replicated genetic material into two daughter cells. Failed cytokinesis can lead to polyploidy. DNA repair defects also promote the occurrence of breakage-fusion-bridge (BFB), a mechanism that accumulates chromosome aberrations through consecutive cell divisions, however, giant cell glioblastoma generally does not exhibit intrachromosomal gains or losses (13). Therefore, the BFB mechanism is not likely to be involved in the formation of giant polyploid cells in giant cell glioblastoma.

Giant cell glioblastoma commonly exhibits two genomic changes: *TP53* mutation and a haploid genome. Interestingly, a subset of B-cell acute lymphoblastic leukemia (B-ALL) patients with a near-haploid genome also carries a germline *TP53* mutation (14). The concurrent occurrence of *TP53* mutation and haploid genome in both giant cell glioblastoma and B-ALL suggests a potential causative association. The haploid genome in cancer cells is thought to result from mitotic catastrophe, leading to the simultaneous loss of multiple suppressor genes, which can drive cancer cell evolution efficiently. However, such a dramatic genomic alteration would typically trigger apoptosis. P53 is a key regulator of cell death in response to genome instability, and its loss of function may help cancer cells evade cell death, even in the presence of mitotic catastrophe (15), thereby facilitating further malignant evolution.

The *PTPRZ1-MET* fusion has been identified in several types of cancer, mostly in glioblastoma and lung cancer (16, 17). The chimeric *PTPRZ1-MET* protein functions as a constitutively activated tyrosine kinase involved in processes such as cell proliferation, survival, and migration (18). *MET*-specific inhibitors, such as Tepotinib and Capmatinib, have been FDA-approved for the treatment of metastatic non-small cell lung cancer (NSCLC) with *MET* exon 14 skipping activation mutations. These inhibitors have shown efficacy *in vitro* against glioblastoma with *PTPRZ1-MET* rearrangement; however, their clinical efficacy in glioblastoma is currently unclear, with ongoing clinical trials investigating their potential.

In conclusion, we present a rare case of giant cell glioblastoma with an inherited germline *CHEK2* mutation. Both CHEK2 and p53 are integral components of the ATM-CHEK2-p53 axis, and the simultaneous occurrence of a germline *CHEK2* mutation and somatic *TP53* mutation in this case strongly suggests the involvement of DNA repair deficiency in the development of this disease.

## Data Availability

The original contributions presented in the study are included in the article/**Supplementary Material**. Further inquiries can be directed to the corresponding authors.
