# Supplementary material for: Case report: Germline CHEK2 mutation is associated with a giant cell glioblastoma
Source: Front Oncol. 2024 Oct 1;14:1361928. doi: 10.3389/fonc.2024.1361928 (PMC11474180; doi:10.3389/fonc.2024.1361928)
Supplement: Supplementary file 1 [file DataSheet1.pdf]

Germline *CHEK2* Mutation is Associated with a Giant Cell Glioblastoma

Yongfeng Bi[1†], Dong Wan[2†], Si Chen[2], Huafei Chen[2], Lingchuan Guo[3], Xiaoshun He[3], Rong Rong[4], Jinyuan Xiao[5], Wei Gao[1*], Sheng Xiao[6*]

Supplementary data:

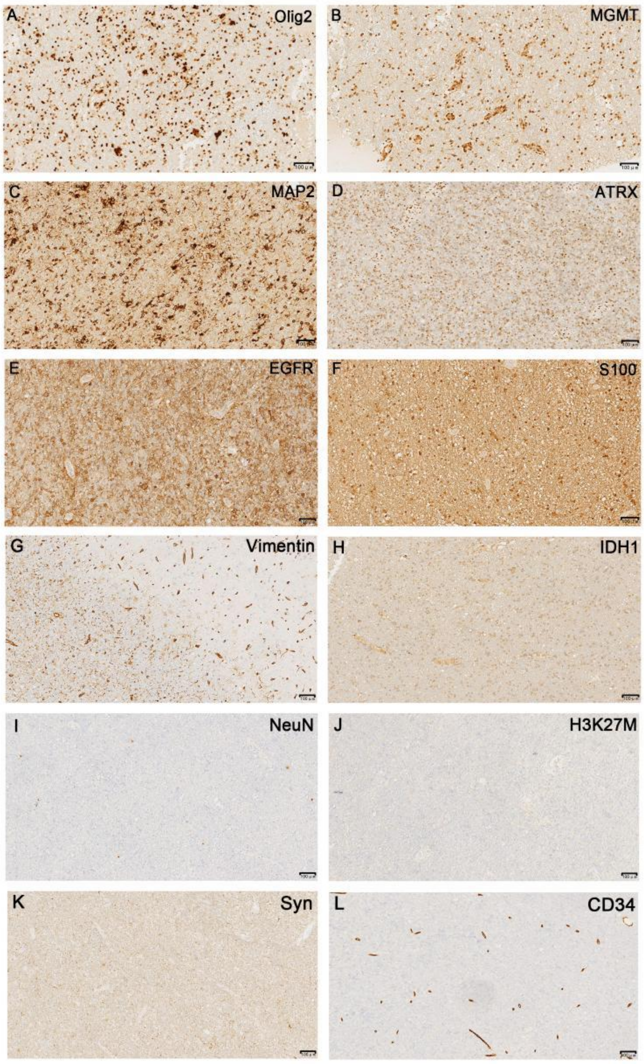

Supplementary Figure 1: Immunohistochemical staining was positive for Olig-2, MGMT, MAP2, ATRX, EGFR, S100, and focal vimentin (A-G) and negative for IDH-1, NeuN, H3K27M, Syn, and CD34 (H-L).

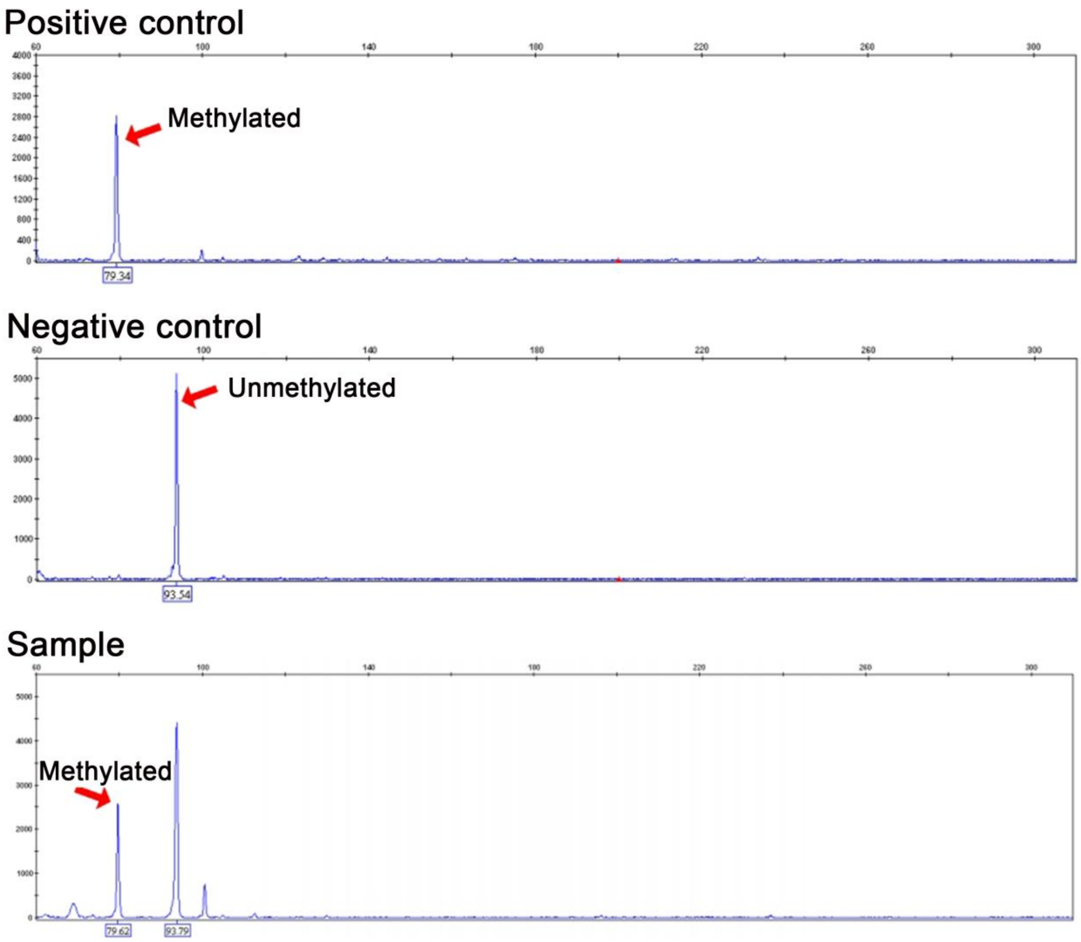

Supplementary Figure 2: Methylation-specific PCR (MSP) and capillary electrophoresis demonstrated methylation of *MGMT*. The capillary electrophoresis fragment of the methylated *MGMT* is 79 bp.

Antibodies	Manufacturers*	Dilution
GFAP	2	1:200
lig-2	3	R**
MGMT	3	R
MAP2	3	R
ATRX	3	R
P53	2	1:200
EGFR	2	1:200
S100	1	R
Vimentin	2	1:200
Ki-67	2	1:100
IDH-1	2	1:100
NeuN	3	R
H3K27M	3	R
Syn	1	R
CD34	1	1:200

Supplementary Table 1：

* 1: DAKO; 2: GeneTech (Shanghai) Company Limited; 3: Beijing Zhongshan Jinqiao Biotechnology Co., Ltd; **R: ready-to-use solution
